# Indian consensus statements on irritable bowel syndrome in adults: A guideline by the Indian Neurogastroenterology and Motility Association and jointly supported by the Indian Society of Gastroenterology

**DOI:** 10.1007/s12664-022-01333-5

**Published:** 2023-03-24

**Authors:** Uday C. Ghoshal, Sanjeev Sachdeva, Nitesh Pratap, Arun Karyampudi, Uzma Mustafa, Philip Abraham, Chetan B. Bhatt, Karmabir Chakravartty, Sujit Chaudhuri, Omesh Goyal, Govind K. Makharia, Manas Kumar Panigrahi, Prasanta Kumar Parida, Sudhanshu Patwari, Rajesh Sainani, Shine Sadasivan, M. Srinivas, Rajesh Upadhyay, Jayanthi Venkataraman

**Affiliations:** 1 Department of Gastroenterology, Sanjay Gandhi Postgraduate Institute of Medical Sciences, Lucknow, 226 014 India; 2 Department of Gastroenterology, GB Pant Hospital, New Delhi, 110 002 India; 3 Department of Gastroenterology, KIMS Hospital, Secunderabad, 500 003 India; 4 Department of Gastroenterology, GSL Medical College and General Hospital, Rajahmundry , 533 296 India; 5 Department of Gastroenterology, P. D. Hinduja Hospital, Mumbai, 400 016 India; 6 Sir HN Reliance Foundation Hospital, Mumbai, 400 004 India; 7 Department of Gastroenterology, Woodland Multispeciality Hospital, Kolkata, 700 027 India; 8 Department of Gastroenterology, AMRI Hospitals, Salt Lake, Kolkata, 700 098 India; 9 Department of Gastroenterology, Dayanand Medical College and Hospital, Ludhiana, 141 001 India; 10 Department of Gastroenterology, All India Institute of Medical Sciences, New Delhi, 110 029 India; 11 Department of Gastroenterology, All India Institute of Medical Sciences, Bhubaneswar, 751 019 India; 12 Department of Gastroenterology, SCB Medical College and Hospital, Cuttack, 753 001 India; 13 Patwari Clinic, Ahmedabad, 380 054 India; 14 Department of Gastroenterology, Jaslok Hospital, Mumbai, 400 026 India; 15 Department of Gastroenterology, Amrita Institute of Medical Sciences, Kochi, 682 041 India; 16 Department of Gastroenterology, Gleneagles Global Health City, Chennai, 600 100 India; 17 Department of Gastroenterology, Max Superspeciality Hospital, New Delhi, 110 017 India; 18 Department of Gastroenterology, Sri Ramachandra Institute of Higher Education and Research, Chennai, 600 116 India

**Keywords:** Bristol stool form scale, COVID-19, Disorders of gut-brain interaction, FODMAP, Functional gastrointestinal disorders, Post-infection IBS, Rome criteria, Small intestinal bacterial overgrowth, Tropical sprue

## Abstract

The Indian Neurogastroenterology and Motility Association (INMA), earlier named the Indian Motility and Functional Diseases Association developed this evidence-based practice guidelines for the management of irritable bowel syndrome (IBS). A modified Delphi process was used to develop this consensus containing 28 statements, which were concerning diagnostic criteria, epidemiology, etiopathogenesis and comorbidities, investigations, lifestyle modifications and treatments. Owing to the Coronavirus disease-19 (COVID-19) pandemic, lockdowns and mobility restrictions, web-based meetings and electronic voting were the major tools used to develop this consensus. A statement was regarded as accepted when the sum of “completely accepted” and “accepted with minor reservation” voted responses were 80% or higher. Finally, the consensus was achieved on all 28 statements. The consensus team members are of the view that this work may find use in teaching, patient care, and research on IBS in India and other nations.

## Introduction

Irritable bowel syndrome (IBS) is a common condition, which leads to significant morbidity, work absenteeism, loss of productivity, and economic burden to the society and impacts the quality of life of patients [1]. There have been several recent developments in the understanding of pathophysiology, diagnosis and treatment of IBS in India [1]. Also, the availability of drugs in India is quite different from the rest of the world [2]. The practicing condition of physicians in India is variable too. Thus, the Indian Neurogastroenterology and Motility Association (INMA), earlier called Indian Motility and Functional Disease Association, was keen on conducting an Indian IBS consensus with the following aims: (a) to provide guidelines to physicians for imparting standard care to patients suffering from IBS; (b) to share knowledge about IBS among doctors and healthcare professionals; and (c) contribute to the advancement of research on IBS. This consensus was also jointly supported by the Indian Society of Gastroenterology.

## Methods

Eighteen consensus team members were chosen from different (eastern, western, northern, and southern) regions of India based on their interest and publications on functional gastrointestinal (GI) disorders, currently called disorders of gut-brain interaction (DGBI), especially IBS. Initially, 37 statements were composed. These statements were emailed to the team members and they were requested to provide their input. The received responses were collated and summarized and uploaded as a document on Google Docs and the team members were invited to edit the statements. Owing to the Coronavirus disease-19 (COVID-19) pandemic, lockdowns, and travel restrictions, face-to-face meetings could not be held for some of the consensus meetings. A Google meet was organized on January 12, 2021, to discuss the statements with the team members; some of the statements were deleted or modified at this stage, resulting in 28 statements in all including diagnostic criteria and epidemiology, etiopathogenesis and comorbidities, investigations, lifestyle modifications and treatments. A modified Delphi method was used in the consensus process.

The statements were posted on Survey Monkey (SurveyMonkey Enterprise, San Mateo, CA, USA) for the first round of voting in February 2021. The second and final voting rounds were delayed due to the second wave of the COVID-19 pandemic and could not be held until the end of May 2021. Subsequently, the results of the consensus were presented to the members of the Indian IBS Consensus team on August 8, 2021, via Google meet.

All members of the consensus team met in Lucknow, India on 7th of May 2022 and finalized the manuscript during the 5th Annual Congress of the INMA (www.gimotilityindia.in), earlier called Indian Motility and Functional Disease Association. A statement was regarded as accepted when the sum of the “completely accepted” and “accepted with minor reservation” votes was 80% or higher. The consensus was achieved on all 28 statements. The responses were collated and the summaries were examined for consensus. As shown in Table 1, the grade of the evidence and the level of agreement were established on the scheme of the Grading of Recommendations Assessment, Development, and Evaluation (GRADE) Working Group [3]. The result of the consensus was presented to the participating members of the INMA and the other delegates during the 5th Annual Congress of the Association (www.gimotilityindia.in) in Lucknow, India, on 7th of May 2022. The levels of evidence were reviewed for which preference was given to studies from India.

**Table 1:** Level of the agreement, level of evidence, and recommendation used in this consensus

Level of agreement	
I	Accepted completely
II	Accepted with minor reservation
III	Accepted with major reservation
IV	Rejected with reservation
V	Rejected completely
Level of evidence	
I	Evidence obtained from at least one randomized controlled trial
II-1	Evidence obtained from well-designed controlled trials without randomization
II-2	Evidence obtained from well-designed cohort or case-controlled study
II-3	Evidence obtained from the comparison between time and places with or without intervention
III	The opinion of respected authorities, based on experience or expert committees
Recommendation (based on the quality of evidence)	
A	There is good evidence to support the statement
B	There is fair evidence to support the statement
C	There is poor evidence to support the statement but recommendation made on other grounds
D	There is fair evidence to refute the statement
E	There is good evidence to refute the statement

## Consensus statements

- **Statement 1:** IBS is a common condition in clinical practice and the Indian community

**Voting summary:** Accepted completely: (100%).

**Level of evidence:** II-2

**Grade of recommendation:** B

Several epidemiological studies showed IBS to be common in the Indian population [4]. An early house-to-house survey in an urban population of Mumbai (2549 presumably healthy adults) primarily evaluating the prevalence of dyspepsia showed a 7.2% prevalence of IBS [5]. Table 2 summarizes the community-based studies on the prevalence of IBS in the Indian population. It shows the prevalence of IBS in the Indian community varies from 0.4% to 4.2% [6–9].

**Table 2:** Prevalence of irritable bowel syndrome in India according to the population-based studies

Author, year	Study setting	Diagnostic criteria	Site	Total number of subjects	Number (%) IBS	Male:female among IBS subjects	Number (%) IBS-C	Number (%) IBS-D	Number (%) IBS-M	Number (%) IBS-U
Sperber et al. [6] 2021	Community	Rome III	Southern India: Telengana Northern India: Uttar Pradesh	4592	18 (0.4)	Data not reported separately	Data not yet reported	Do	Do	Do
Ghoshal and Singh [7] 2017	Community	Rome III	Jaunpur District, Uttar Pradesh	2774	75 (2.7)	45:30	5/75 (6.6)	8/75 (10.6)	0	62/75 (82.6)
Makharia et al. [8] 2011	Community	Rome III	Ballavgarh, Haryana	4767	191 (4)	77:114	12/191 (6.3)	72/191 (37.7)	81/191 (42.4)	26/191 (13.6)
Ghoshal et al. [9] 2006	Convenient sample	Manning 3	Pan-Indian study	4500	189 (4.2)	109:80	Not reported	Not reported	Not reported	Not reported

*IBS* irritable bowel syndrome, *IBS-C* constipation dominant irritable bowel syndrome, *IBS-D* diarrhea dominant irritable bowel syndrome, *IBS-M* mixed irritable bowel syndrome, *IBS-U* undefined irritable bowel syndrome

The reason for the very low prevalence of IBS in the recent global study might be related to the design of the study [6]. This study included only one member per family during the household survey even though this disorder shows clustering within the family. Hence, it might have underestimated the disease burden. Another reason could be related to the fact that overlap disorders, which is the commonest type of functional gastrointestinal disorder (﻿FGID), might result in under-estimation of the disease burden of pure disorders [10]. This study also showed that household survey generally records a lower prevalence than internet survey possibly due to recruitment bias in the latter method. It is important to note that in India, a household survey was conducted in contrast to the Western countries. Rome IV criteria underdiagnose IBS more than the Rome III criteria [6, 11]. Despite these methodological issues, the lower prevalence of IBS in India is quite noteworthy. It may be related to dietary, socio-cultural, gut microbiota and hygiene hypothesis-related factors.

- **Statement 2:** IBS is as prevalent in the male as the female population in India

**Voting summary:** Accepted completely: (88.87%), accepted with minor reservation: (11.11%).

**Level of evidence:** II-2

**Grade of recommendation:** B

IBS is more prevalent among females than males in the West [12]. Many Indian clinic studies showed that more than two-thirds of IBS patients in Indian studies are male [9]. A possibility of referral bias due to male patients more often seeking healthcare facilities, particularly in the advanced centers from where studies are published has been considered [9]. However, even in the community studies, the female preponderance was either lacking or the ratio of male to female was quite close (Table 2). Hence, it may be concluded that male subjects also suffer from IBS quite commonly.

- **Statement 3:** IBS patients in India often have other overlapping FGIDs

**Voting summary:** Accepted completely: (100%).

**Level of evidence:** II-2

**Grade of recommendation:** B

Earlier, it was thought that FGIDs (currently called DGBI) are isolated disorders without any overlap. However, several studies from Asia, including India, showed that the overlaps between various FGIDs are common rather than exceptions [7, 10]. In an epidemiological study from northern India, of 2774 subjects 413 (14.9%) had dyspepsia alone, 75 (2.7%) IBS alone and 115 (4.1%) had dyspepsia-IBS overlap, respectively by Rome III criteria [7]. Another study by Rome IV criteria in northern India showed functional dyspepsia-IBS overlap in 4.4% of the 1309 subjects [13]. In a recent multicentric study by the Rome Foundation, patients with multiple FGIDs were found to have more psychological comorbidity, healthcare utilization and IBS severity [14]. It must be emphasized that owing to busy work schedules, the overlaps may often be overlooked by physicians, particularly in India and the adjoining nations, as they may primarily focus on predominant symptoms instead of directing adequate attention to recognize the overlaps in their clinical practice [15, 16]. To address these lacunae, it was felt imperative to develop, translate and validate diagnostic questionnaires into regional languages, which helped in the diagnosis of otherwise missed FGID overlaps [15, 16]. Table 3 presents the IBS-FGID overlap in studies conducted entirely or partly in India [5, 7, 13, 15].

**Table 3:** The overlap of irritable bowel syndrome and the other functional gastrointestinal disorders in studies conducted partly or entirely in India

Reference	Location	Sample size	Defining criteria	FD	IBS	FGID overlap
Shah et al. [5], 2001	India	2549 presumably HS	FD: upper abdominal pain/discomfort for at least one month IBS: Manning	26.0%	3.1%	FD-IBS (4.3%)
Rahman et al. [15], 2016	India, Bangladesh	40 IBS	Rome III/EAR3Q	30.0% (1st visit); 30.0% (follow-up visit)	All 40 patients had IBS	IBS-FD
Rahman et al. [15], 2016	India, Bangladesh	35 FD	Rome III/EAR3Q	All 35 patients had FD	22.8% (1st visit); 28.5% (follow-up visit)	FD-IBS
Ghoshal and Singh [7], 2016	India	2774	Rome III	14.9%	2.7%	FD-IBS (4.1%)
Goyal et al. [13], 2021	India	1309	FD: Rome IV; IBS: Rome III	15.2%	6.2%	FD-IBS (4.4%)

*EAR3Q* enhanced Asian Rome III questionnair; *FGID* functional gastrointestinal diseases, *FD* functional dyspepsia, *IBS* irritable bowel syndrome, *HS* healthy subjects

- **Statement 4:** Etiopathogenesis of IBS is multi-dimensional including gut-specific mechanisms, altered gut-brain interaction, food intolerance, psychosocial and genetic factors

**Voting summary:** Accepted completely: (100%).

**Level of evidence:** II-2

**Grade of recommendation:** B

The multifactorial pathogenesis of IBS has been reviewed recently [17]. These factors include (i) peripheral factors such as abnormal GI motility, GI inflammation and altered gut permeability, luminal microenvironment alteration including gut microbiota dysbiosis and small intestinal bacterial overgrowth (SIBO), host-microbe interaction, bile acid malabsorption, pathogenic infection, dietary factors and neurohumoral dysregulation including altered serotonergic transmission and visceral hypersensitivity and (ii) central factors (psychological stress, cognitive dysfunction, abnormal emotional arousal system response, and sleep dysfunction). Genetic factors may underlie peripheral and central pathophysiological mechanisms.

- **Statement 5:** Patients with IBS, particularly those with diarrhea-predominant IBS (IBS-D), are more likely to have SIBO and gut dysbiosis

**Voting summary:** Accepted completely: (72.22%), accepted with minor reservation: (27.78%).

**Level of evidence:** II-1

**Grade of recommendation: **A

A recent meta-analysis, which included at least four Indian studies, showed that 36.7% of IBS patients had a positive test for SIBO [18]. Patients with IBS were 2.6 and 8.3 times more likely to have a positive test for SIBO as compared with healthy controls using glucose hydrogen breath test﻿ (GHBT) and jejunal aspirate culture, respectively [18]. Patients with IBS-D were more likely to have positive GHBT as compared with the other subtypes [18]. Somewhat similar results have been found in another meta-analysis [19].

- **Statement 6:** Excessive methane production slows gut transit and is associated with constipation-predominant IBS (IBS-C)

**Voting summary:** Accepted completely: (77.78%), accepted with minor reservation: (22.22%).

**Level of evidence:** I

**Grade of recommendation:** B

A meta-analysis showed that excess breath methane during lactulose or glucose hydrogen breath test was associated with chronic constipation [20]. A few Indian studies showed patients with chronic constipation (functional constipation and IBS-C) had a greater amount of breath methane than non-constipating IBS [21, 22]. On treatment with rifaximin, constipation improved, breath methane reduced and colon transit improved as compared to treatment with placebo [21]. Two other studies from the USA, one prospective and the other retrospective, also supported the findings of this study [23, 24]. More studies are needed on this issue.

- **Statement 7:** Gastrointestinal infection with varied pathogens may result in post-infection IBS (PI-IBS)

**Voting summary:** Accepted completely: (100%).

**Level of evidence:** II-1

**Grade of recommendation:** A

Of the multiple factors involved in the pathogenesis of IBS (Table 4) [21,22,27-53], development of the condition following acute infectious gastroenteritis is perhaps the strongest proof of micro-organic basis of a subset of these patients. Multiple studies from all over the World showed that following GI infection with bacterial, protozoal and viral pathogens may be followed by PI-IBS [25]. Though infection and infestations are common in developing countries, including India, the studies on the development of PI-IBS from India are scanty. Of the four available studies on this issue from the Indian authors, one was entirely performed in a population in Bangladesh [26], a country with similar socio-cultural factors and environment (Table 4) [26, 37–39]. Two of the recent studies were done among patients with COVID-19 [37, 38]. All the studies showed that the development of PI-IBS was common following acute GI infection and COVID-19. More studies from India are needed on this issue.

**Table 4:** Studies on pathophysiological mechanisms of irritable bowel syndrome either entirely or partly from India

Pathophysiological factor	Author, year	Number of subjects	Findings
Slow colon transit (methane)	Ghoshal et al. [22], 2016	47 IBS (20 IBS-C), 30 HC	*Methanobrevibacter smithii* copy number was higher among IBS than HC, and IBC-C > IBS-D *M. smithii* copy number negatively correlated with the stool frequency Breath methane levels correlated with *M. smithii* loads
	Ghoshal et al. [21], 2018	23 CC (10 IBS-C), 68 non-C- IBS	CC patients tended to be methane producer more often (56.5% vs. 36.5%) and had greater AUC for methane Rifaximin reduced AUC for methane more than placebo, normalized CTT in 66.7%, improved weekly stool frequency
	Rana et al. [27], 2009	345 IBS, 254 HC	Prevalence of predominant methanogenic flora in IBS and HC was 14.5% and 34.6%
Fecal evacuation disorder	Goyal et al. [28], 2019	132 FC and 99 IBS-C, 30 HC	FED was diagnosed in 55.3% and 46.5% of FC and IBS-C vs. 3.3% in HC
	Ghoshal et al. [29], 2016	249 CC	34% had FED; Rome III criteria for IBS were equally fulfilled by patients with and without FED (89% vs. 81.2%)
	Shah et al. [30], 2014	74 FC, 25 IBS-C	Dyssynergic defecation -38% vs. 48% in FC, IBS-C STC-15% vs. 16% in FC, IBS-C
Gut microbiota dysbiosis	Shukla et al. [31], 2015	47 IBS, 30 HC	Relative copy number of Bifidobacterium was lower, while those of *Ruminococcus productus*-*Clostridium coccoides*, *Veillonella*, *Bacteroides thetaiotamicron*, *Pseudomonas aeruginosa*, and Gram-negative bacteria were higher among IBS patients than HC
	Rana et al. [32], 2008	225 IBS, 100 HC	Frequency of SIBO by GHBT was higher (11.1%) in IBS than HC (1%)
	Rana et al. [33], 2012	175 IBS-D, 150 HC	SIBO positivity in patients (34.3% by LHBT, 6.2% by GHBT) while in HC (30%, 0.66%)
	Ghoshal et al. [34], 2010	129 IBS, 73 with chronic non-specific diarrhea (CNSD), 51 HC	SIBO positivity by GHBT: 21.9% in CNSD, 8.5% in IBS, 2% in HC
	Sachdeva et al. [35], 2011	59 IBS, 37 HC	SIBO (by GHBT) was more frequent in IBS than HC (23.7% vs. 2.7%)
	Ghoshal et al. [36], 2014	80 IBS, 10 historical controls	19% IBS and 0% controls had SIBO by upper gut aspirate culture
Post-COVID-19 IBS	Ghoshal et al. [37], 2022	280 COVID-19, 264 HC	At 6-month follow-up, one (0.37%) HC developed IBS, while among COVID-19, 15 (5.3%) had IBS, and 5 (1.8%) had IBS-dyspepsia overlap
	Golla et al. [38], 2022	320 COVID-19, 600 HC	At 3-month follow-up, none of HC developed IBS, while among COVID-19, 8 (2.5%) had IBS and 2 (0.6%) had IBS-dyspepsia overlap
Post-infection IBS	Rahman et al. [26], 2018	345 patients with AGE, 345 HC	AGE patients more often developed PI-IBS than HC (16.5 vs. 2.6%)
	Parida et al. [39], 2019	136 hospitalized AGE patients	35 (25.7%) developed PI-IBS after 6 months; younger age, long duration of AGE, and abdominal pain were independent risk factors for PI-IBS
Celiac disease/gluten sensitivity	Zanwar et al. [40], 2015	60 IBS-randomized, double blinded placebo-controlled trial (30 gluten, 30 placebo)	Post-4 weeks of food rechallenge, gluten group exhibited worsening of symptoms, compared to the placebo group
	Sharma et al. [41], 2015	362 IBS	22 (6.1%) had positive anti-tTG antibody (3 [0.8%] CeD and 19 potential CeD), IgA AGA was positive in 104 (28.7%) suggestive of gluten sensitivity
FODMAP intolerance	Goyal et al. [42], 2021	101 IBS-D randomized to LFD and TDA	Significant improvement in symptoms and quality of life in the LFD group
Fructose malabsorption	Sharma et al. [43], 2014	97 IBS, 41 HC	IBS more often had FM than HC (14.4% vs. 2.4%). Patients with FM more often had IBS-D
Lactose malabsorption	Gupta et al. [44], 2007	124 IBS, 53 HC	Lactose intolerance (abnormal lactose hydrogen breath test or LTT) was comparable among IBS patients and HC (82% vs. 77%)
	Rana et al. [45], 2001	25 IBS (11-D-IBS), 25 HC	Lactose hydrogen breath test positivity higher in IBS-D than HC, but comparable in non-D-IBS vs HC
Lactose malabsorption	Ghoshal et al. [46], 2009	192 IBS	65% and 71% were lactose HBT and LTT positive, respectively
	Kumar et al. [47], 2012	150 IBS, 252 HC	C/T-13910 and G/A-22018 polymorphisms of lactase gene were comparable among IBS and HC, but these were more common among D-IBS
Inflammation/immune activation	Shukla et al. [48], 2018	47 IBS, 25 HC	The mRNA levels of TLR-4, TLR-5, and IL-6 were upregulated in IBS patients than controls; IBS-D had lower mRNA levels of IL-10
	Patel et al. [49], 2017	51 IBS, 29 HC	Levels of IL-2 and IL-8 were significantly higher in IBS than HC; levels were higher in PI-IBS than non-PI-IBS
	Srivastava et al. [50], 2014	221 IBS, 273 HC	Genotype 1/1 (IL-1RA overproducer) was less frequent among IBS than HC, while genotypes 1/3 and 2/3 were higher in IBS
	Rana et al. [51], 2012	63 IBS-D, 62 HC	Levels of IL-6 and TNF-alpha higher in IBS-D than in HC
	Aggarwal et al. [52], 2018	30 IBS-D, 29 HC	Diminished level of GABA in IBS-D compared to HC; GABA reduced the expression of proinflammatory cytokines in LPS stimulated HT-29 cells
Disordered gut-brain interaction	Guleria et al. [53], 2017	20 IBS, 10 HC	On fMRI, brain response to rectal balloon distension differed among IBS and controls, and among patients with IBS-C and IBS-D

*IBS* irritable bowel syndrome, *SIBO* small intestinal bacterial overgrowth, *COVID-19* coronavirus disease-19, *FODMAP* fermentable oligosaccharides, disaccharides, monosaccharides and polyols, *HC* healthy controls, *CC* chronic constipation, *FC* functional constipation, *IBS-C* irritable bowel syndrome with predominant constipation, *IBS-D* irritable bowel syndrome with predominant diarrhea, *IBS-U* irritable bowel syndrome unclassified, *AUC* area under curve, *CTT* colonic transit time, *FED*, fecal evacuation disorder, *STC* slow transit constipation, *LHBT* lactulose hydrogen breath test, *GHBT* glucose hydrogen breath test, *AGE* acute gastroenteritis, *CeD* celiac disease, *AGA* antigliadin antibody, *LFD* low-FODMAP diet, *TDA* traditional dietary advice, *FM* fructose malabsorption, *PI-IBS* post-infection irritable bowel syndrome, *LPS* lipo-polysaccraride, *fMRI* functional magnetic resonance imaging, *LTT* lactose tolerance test, *TLR* toll-like receptor, *RNA* ribonucleic acid, *IL* interleukine

- **Statement 8:** A diagnosis of PI-IBS by Rome criteria does not exclude underlying post-infectious malabsorption syndrome (tropical sprue)

**Voting summary:** Accepted completely: (66.67%), accepted with minor reservation: (22.22%), rejected with major reservation: (11.11%).

**Level of evidence:** II-2

**Grade of recommendation:** B

Following an attack of acute infectious gastroenteritis, though 90% of patients recover completely, the remaining may continue to have chronic GI symptoms such as loose motion, abdominal pain or discomfort, bloating, etc., beyond six months fulfilling the Rome criteria for IBS [54]. Earlier, it was reported that the patients continuing to have frequent and loose stools, on investigations, may be found to have mucosal malabsorption such as abnormal d-xylose test (denoting carbohydrate malabsorption), fecal fat (denoting fat malabsorption) and abnormal mucosal histology [55]; these patients improve following treatment with antibiotics and vitamin supplementation (folic acid, vitamin B_12_). The latter condition is known as post-infectious malabsorption syndrome, popularly called tropical sprue. There is considerable overlap in the clinical presentations of the patients with PI-IBS and tropical sprue [54]. Since hardly any study published earlier on PI-IBS investigated patients for malabsorption syndrome, the exact frequency of tropical sprue in patients with PI-IBS was not known. However, in a recent study, about 10% of patients with PI-IBS undergoing work-up were found to have tropical sprue despite fulfilling Rome criteria for IBS [26]. In another recently published study among patients with post-COVID-19 IBS, mucosal malabsorption documented by abnormal d-xylose test alone was present in 30% and by two abnormal tests (abnormal d-xylose test and low serum vitamin B_12_) in 4% of patients [38]. A study from the USA also showed the occurrence of both PI-IBS and tropical sprue following acute gastroenteritis among army personnel posted in the Iraq war suggesting a common link between the two conditions [56]. Hence, it is important to investigate patients with PI-IBS for tropical sprue. This has also been recommended by the Rome PI-IBS committee [57]. More studies are needed on this issue.

- **Statement 9:** COVID-19 may lead to post-COVID-19 IBS

**Voting summary:** Accepted completely: (27.78%), accepted with minor reservation: (61.11%), accepted with major reservation: (5.56%), rejected with major reservation: (5.56%).

**Level of evidence:** II-2

**Grade of recommendatio**n: B

Based on the shared mechanistic shreds of evidence, a possibility of the development of PI-IBS following COVID-19 has been suggested [58]. These mechanistic shreds of evidence include the presence of angiotensin-converting enzyme-2 (ACE-2) receptors in the GI tract resulting in infection of the GI tract by the virus, the presence of severe acute respiratory syndrome coronavirus-2 (SARS-CoV-2) ribonucleic acid (RNA) in the stool of these patients, presence of GI symptoms, including diarrhea, in about 10% to 20% patients with COVID-19, raised fecal calprotectin and mucosal serotonin, macroscopic and histological evidence of GI mucosal injury, increased mucosal permeability, gut microbiota dysbiosis, involvement of the nervous system including the enteric nervous system by the virus, and increased stress due to the pandemic [58]. In the world’s first case–control study on post-COVID-19 FGID reported from India and Bangladesh, at six months following COVID-19, IBS, dyspepsia and their overlap developed in 5.3%, 2.1%, and 1.8% patients, respectively [37]; these figures were significantly greater than in the control population [37]. The presence of anosmia, ageusia, chronic bowel dysfunction, dyspeptic symptoms at one and three months and psychological comorbidity were predictors for the development of post-COVID-19 FGID (now called DGBI) [37]. In the second study on this from Georgia, USA, of the 164 of 1114 subjects with COVID-19 who participated in follow-up study, 108 (66%) fulfilled the criteria for at least one DGBI [59]. Such a high frequency of development of post-COVID-19 DGBI in that study might be related to recruitment bias as only 164 of 1114 subjects participated in the follow-up study. Two other studies published recently, one from India and the other multicentric (GI-COVID-19 study), showed the occurrence of post-COVID-19 FGID (now called DGBI) after the occurrence of COVID-19 [38, 60]. In the Indian study, the frequency of post-COVID-19 IBS during six-month follow-up was 7% [38]. In the other multicentric study, 3.2% of hospitalized patients with COVID-19 developed IBS by Rome IV criteria during 12-month follow-up [60].

- **Statement 10:** Psychological and somatoform comorbidities are common in IBS

**Voting summary:** Accepted completely: (94.44%), accepted with minor reservation: (5.56%).

**Level of evidence:** II-2

**Grade of recommendation:** B

Psychological comorbidities, including anxiety and depression, are common in IBS patients both globally and India [61, 62]. As evidenced by the voting summary, the importance of psychological factors is apparent from a cent percent agreement among experts; this is quiet close to the agreement in the second Asian IBS consensus [63]. In a recent meta-analysis on seven case–control studies including 590 patients with IBS and 1520 controls from India, the pooled odds ratios of anxiety and depression were 8.060 (95% confidence interval [CI] 4.007–16.213) and 7.049 (95% CI 3.281–15.147) compared to controls by random effect models, respectively [62]. There was significant heterogeneity in the included studies [62]. Moreover, most studies were from tertiary urban centers, posing the possibility of recruitment bias overestimating the frequency.

- **Statement 11:** Dietary FODMAPs (fermentable oligo-, di-, monosaccharides and polyols) may contribute to symptoms of IBS

**Voting summary:** Accepted completely: (88.89%), accepted with minor reservation: (11.11%).

**Level of evidence:** I

**Grade of recommendation:** B

Mechanistically, high-FODMAP foods cause symptom exacerbation in IBS patients [64–66]. A randomized trial from northern India showed that a low-FODMAP diet mitigated GI symptoms (frequency of abdominal pain, abdominal distention, bowel habit satisfaction) and improved quality of life and resulted in a significant reduction in the need for medication in IBS-D patients [42].

Milk, which contains lactose, is one of the high-FODMAP foods. The frequency and degree of lactose malabsorption are higher in southern than in northern Indian healthy populations [67]. Though the frequency of lactose malabsorption was comparable among patients with IBS as that among healthy controls, the former group exhibited more symptoms than the latter (47.4% compared with 15.6%; *p* = 0.001) [44, 67]. Extrapolating these results, it may be assumed that dietary FODMAPs may contribute to symptom development in more vulnerable groups such as IBS patients. The patients with IBS also had malabsorption of fructose more often by fructose hydrogen breath test, which is another component of high-FODMAP foods, than controls ([14.4% vs. 2.4%]; *p* = 0.04). Patients with IBS-D more often had fructose malabsorption than those with other subtypes of IBS (91% vs. 56%) [43].

Since dietary practices in different regions of India is quite heterogeneous (prevalence of vegetarianism/non-vegetarianism, preference for wheat vs. rice as staple cereal, volumes of milk/milk product consumption), the contribution of dietary FODMAPs as a symptom driver in patients with IBS may be regionally stratified [68]. Owing to regional food preferences and differences in vegetarianism, the northern Indian diet is highest in FODMAP content, followed by central and western Indian diets. The northeastern Indian diet is the lowest in FODMAP content, followed by the southern and eastern Indian diet, which is low in FODMAP [68]. Thus, IBS patients in different regions in India may have a varying predisposition to symptom development due to dietary FODMAPs [68]. The World Gastroenterology Organization provides guidelines to provide clinical practice recommendations on a low-FODMAP diet [69].

- **Statement 12:** Diagnosis of IBS is primarily clinical and based on well-defined symptom-based criteria

**Voting summary:** Accepted completely: (83.33%), accepted with minor reservation: (16.67%).

**Level of evidence:** II-2

**Grade of recommendation:** A

The diagnosis and classification of IBS are based on symptoms, with the need for investigation only when otherwise indicated [70]. The symptoms in patients with IBS may also overlap with those of other FGIDs (overlap disorders) and psychosocial issues, which pose a challenge to correctly diagnose and treat the condition. Diagnosis and management of these overlap disorders have been recently addressed in an Asia–Pacific Consensus [10]. There is no specific biomarker for the diagnosis of IBS to date.

IBS is diagnosed by clinical criteria by a constellation of symptoms without an organic disease explaining these symptoms [63, 70]. Several criteria have been formulated for the diagnosis of IBS, such as Manning, Rome (I, II, III, IV), and Asian criteria (Table 5) [1, 63, 70]. A multicentric Indian IBS study comparing various criteria found that Manning criteria are more sensitive for IBS diagnosis as compared to Rome I, II, and III criteria in the Indian population [71]. The Asian criteria proposed by the Asian Neurogastroenterology and Motility Association, though were better than the Rome I, II, and III criteria, performed worse than the Manning criteria [71]. Several studies did show that fulfilling Rome criteria were associated with a positive diagnosis of IBS without the presence of obvious organic diseases in most of the patients [72]. A systematic review reports that among patients meeting symptom-based criteria for IBS, the probability of organic disease is less than 1% [72]. A house-to-house survey using an enhanced Asian Rome III questionnaire, endoscopy tests and molecular genotyping techniques in the Bangladeshi population, inferred that most people suffered from functional dyspepsia (a common FGID) [73]; of them, 114 of 547 (20.8%) undergoing upper GI endoscopy had organic lesions that could explain their dyspeptic symptoms [73]. Since the epidemiological profile of Indian patients with FGIDs, including IBS, is somewhat similar to the Bangladeshi population [74], it sounds reasonable to conclude that fulfilling symptom-based Rome criteria may be associated with an organic diagnosis in a similar frequency.

**Table 5:** Different symptom-based criteria used for the diagnosis of irritable bowel syndrome

		Manning criteria (any 4 listed symptoms irrespective of duration)	Rome III criteria (any 2 listed symptoms associated with duration)	Asian criteria (any one or more listed symptoms associated with duration)	Rome IV criteria (any 2 or more listed symptoms associated with duration)
	Duration and frequency of abdominal pain/discomfort	Duration not considered	At least 3 months with onset at least 6 months previously; at least 3 days per month	At least ≥ 3 months	Recurrent abdominal pain, on average, at least 1 day per week in the last 3 months. Discomfort as a defining symptom removed.
**Symptoms**	Pain/discomfort in relation to bowel movement	Relief with bowel movement	Improvement with defecation	Relief with defecation	Related to defecation (may decrease or increase)
	Pain in relation to stool type	Pain associated with looser stools	Onset associated with change in form of stool	Change in stool form	Change in the form of stool
	Pain in relation to stool frequency	Pain associated with more frequent stools	Onset associated with change in stool frequency	Change in stool frequency	Change in the frequency of stool
	Fecal evacuation	Sensation of incomplete evacuation			
	Mucus	Passage of mucus			
	Bloating	Abdominal distension

- **Statement 13:** Rome III criteria may be preferred over Rome IV to diagnose IBS in India due to its higher sensitivity

**Voting summary:** Accepted completely: (72.22%), accepted with minor reservation: (27.78%).

**Level of evidence:** II-2

**Grade of recommendation:** B

**Fig. 1:**
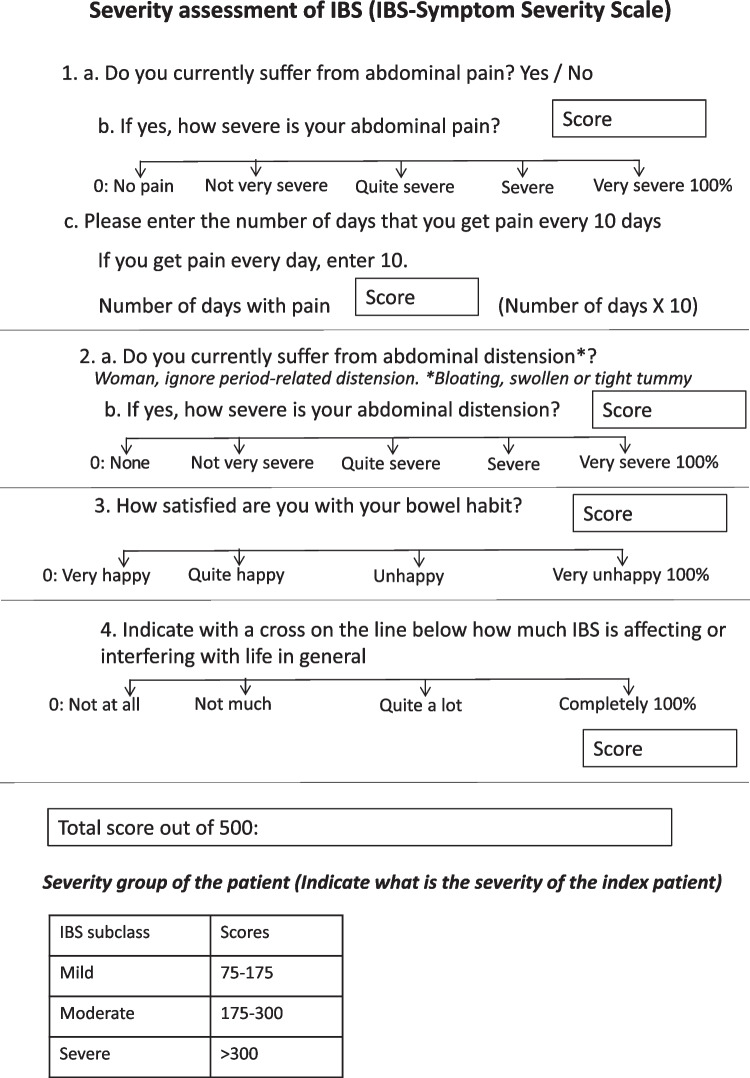
Irritable bowel syndrome (IBS) – symptom severity scale (adapted from Francis et al. [79])

In a multinational study, the prevalence of IBS was lower as per Rome IV criteria than the Rome III criteria all over the World [6]. The frequency of IBS in the internet survey countries by Rome III and IV criteria was 10.1% vs. 4.1% and in the household survey countries was 3.5% vs. 1.5%, respectively [6]. It is important to mention that in India, a household survey was done on the urban and rural populations in the southern and northern Indian populations. In a cross-sectional study in a primary care setting in Malaysia, the frequency of IBS was reduced from 4% by Rome III criteria to 0.8% by Rome IV criteria [75]. Since in the Rome IV criteria for diagnosis of IBS, the frequency threshold of symptoms is based on the studies from the Western population [76, 77] and the presence of abdominal pain has been made mandatory, Rome IV criteria have become quite insensitive worldwide, more so in India. Abdominal pain is less frequent and severe in the Indian population [9, 71]; abdominal bloating and discomfort, which are not included as IBS-defining symptoms according to Rome IV criteria, are frequent in Indian patients with IBS. The natural history and health impact of Rome IV criteria-defined IBS is more severe in comparison to that diagnosed by Rome III criteria [78]. The severity of IBS can be assessed by subjective quantification of the symptoms, their frequency and interference with the life in general (Fig. 1) [79]. This is called IBS symptom severity scale (IBS-SSS) (Fig. 1) [79]. 

Rome III criteria may be preferred over Rome IV to diagnose IBS in India due to its higher sensitivity. However, Manning and Asian criteria were found to be even superior to Rome III criteria [71]. A recent Indian study did show that IBS was less often diagnosed when Rome IV criteria rather than Rome III criteria were used (6.2% vs. 9.5%, respectively) [13]. It appears that Rome IV criteria led to an internal shift in various diagnostic categories of FGID as there was a higher frequency of diagnosis of functional diarrhea and functional constipation than IBS on application of Rome IV criteria [13].

- **Statement 14:** Rome’s subtyping of IBS into constipation and diarrhea-predominant conditions frequently leads to a large proportion of patients remaining unclassified in India

**Voting summary:** Accepted completely: (77.78%), accepted with minor reservation: (22.22%).

**Level of evidence:** II-2

**Grade of recommendation:** B

Application of Rome criteria concerning stool frequency and stool type to stratify IBS patients into IBS-C and IBS-D subtypes often leads to a large proportion of patients being unclassified in India [71]. As reported by a comparison of diagnostic criteria, up to 77.6% of patients could not be classified when the Rome criteria of stool frequency for IBS-C (< 3 stools/week) were adhered to, and 15.7% of patients remained unclassified when Rome criteria of stool form for IBS-C (Bristol stool form 1, 2) were used [71]. Stool form-based feco-graphical analysis led to 16/51 (31.4%) remaining unclassified as either IBS-C/IBS-D [80]. Baseline clinical characteristics of “IBS only” patients in several studies show that a significant proportion of them remain unclassified: 62/75 (82.7%) [7], 7/47 (14%) [31], 17/70 (25%) [43], 16/160 (10%) [81]. The proportion of patients remaining unclassified was also high in IBS overlapping with other FGIDs: IBS-dyspepsia overlaps 72/115 (62.6%) [7]. Comparatively, the proportion remaining unclassified in IBS patients was more by Rome III (9/124 [7.3%]) than by Rome IV (1/81 [1.2%]) criteria [13].

- **Statement 15:** Bristol stool form scale (BSFS) along with other symptoms such as straining, incomplete evacuation, urgency and patient-reported bowel pattern should be assessed to evaluate bowel movement

**Voting summary:** Accepted completely: (94.44%), accepted with minor reservation: (5.56%).

**Level of evidence:** II-2

**Grade of recommendation: **B

In Asian consensus, Bristol stool forms 1–3 have been defined as constipation and 5–7 as diarrhea. Sub-classification of IBS, according to the bowel pattern, could be based on a modification of the Rome III criteria [82]. The BSFS demonstrated substantial validity and reliability [9, 83]. The Asian consensus on IBS suggested that in addition to Bristol types 1 and 2 stool, type 3 stool should also be considered as constipation in Asia [63, 82]. In a multicentre study from India, improvement in subtyping IBS using BSFS as suggested in the Asian consensus has been reported [71]; in this study, applying stool types 3 (as hard stool) and 5 (as a soft stool) as abnormal stool forms allowed more patients to be subtyped compared to the use of Rome subtyping system that considers type 3 to type 5 stools as normal stools [71]. Other constipation-associated symptoms such as straining, feeling of incomplete evacuation, patients’ perception and infrequent bowel movements (less than three bowel movements per week) should also be considered while sub-classifying IBS.

- **Statement 16. A:** In the absence of alarm features, a few baseline investigations are suggested for patients with suspected IBS.
- **Statement 16. B:** The presence of alarm features necessitates diagnostic tests to rule out organic disease.

**Voting summary:** Accepted completely: (100%)

**Level of evidence:** II-2

**Grade of recommendation:** B

**Fig. 2:**
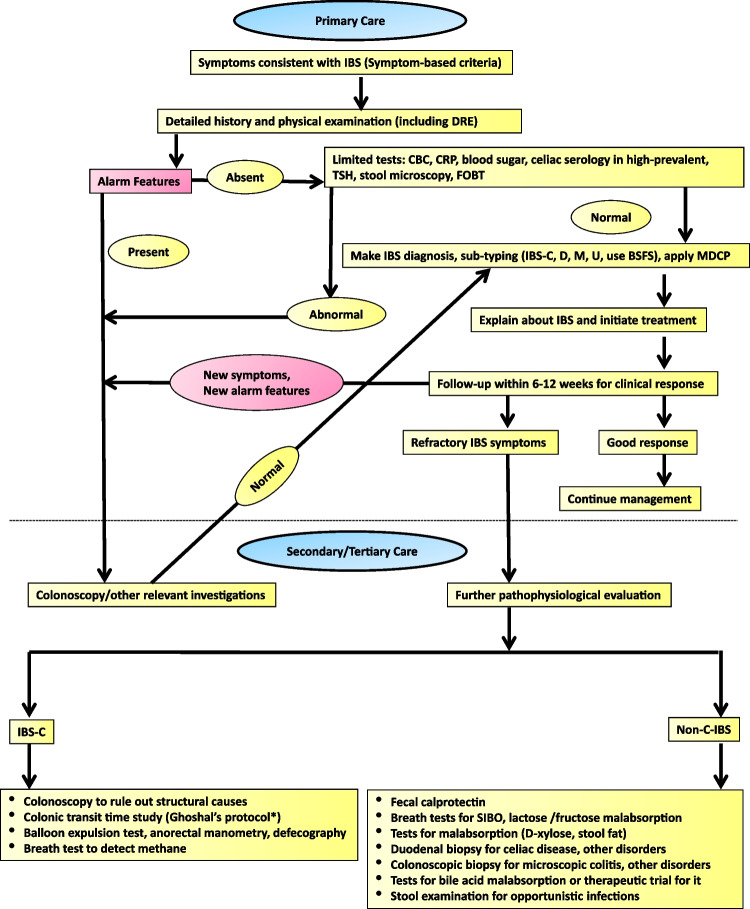
Algorithm of investigation of patients with irritable bowel syndrome. *IBS* irritable bowel syndrome, *DRE* digital rectal examination *CBC* complete blood count, *CRP* C-reactive protein, *TSH* thyroid stimulating hormone, *FOBT* fecal occult blood test, *IBS-C* *D* *M* *U* constipation-, diarrhea-predominant, mixed, unclassified, *BSFS* Bristol stool form scale, *MDCP* multi-dimensional clinical profile *SIBO* small intestinal bacterial overgrowth

Though the yield of investigating patients to exclude organic disease after a criteria-based diagnosis of IBS in the West has been low, due to a lack of high-quality evidence from India and frequent occurrence of GI infection and infestation, a few routine investigations to exclude organic disorders and infection and infestations may be recommended at present (Fig. 2). In the presence of alarm features, which include the onset of symptoms at an age older than 45 years, presence of anemia, blood in the stool, unintended weight loss, nocturnal symptoms, fever, abdominal mass and family history of colorectal cancer, thorough investigations to rule out organic disease are essential. However, it is well recognized that alarm features per se have a low yield of organic disease [84]. However, by consensus, guidelines have adopted these symptoms to focus and fast-track tests for such patients, in systems where healthcare has long waiting times. There is one large cross-section study on this issue [85]. Several studies from other parts of the World including the one mentioned above have looked at the only prevalence of organic disease in those labeled as IBS [86, 87]. A meta-analysis of 28,630 patients with possible IBS who underwent colonoscopy showed a pooled prevalence of colorectal cancer, inflammatory bowel disease and microscopic colitis to be 0.78%, 4.48%, and 2.35%, respectively [88]. The second Asian consensus on IBS also suggested that the shreds of evidence supporting the statement on the predictive value of alarm symptoms to exclude organic disorders are somewhat low [63].

Most studies from India on predicting the utility of alarm symptoms to suggest organic diseases are not undertaken among patients with IBS but have been done on another common FGID, namely functional dyspepsia. In a study from southern India on 900 patients with dyspepsia, upper GI endoscopy revealed benign organic lesions in 38% and malignancy in 5.5% [89]. The authors concluded that the optimal age to begin screening for malignancy in dyspepsia patients in India seems to be 46.5 years [89]. In another recent study from northern India on 294 patients with dyspepsia, the authors found that even among patients younger than 60 years with alarm features, a considerable number of patients had organic lesions (65%) including malignancy (16%) on upper GI endoscopy [90]. These authors chose a cut-off value of 60 years for age as the American College of Gastroenterology (ACG) and Canadian Association of Gastroenterology (CAG) guidelines did not recommend upper GI endoscopy to investigate alarm features for dyspepsia patients under the age of 60 to exclude upper GI malignancy [91]. The cut-off value of some of the alarm features such as age may differ in different populations. There is no Indian study on this issue concerning IBS. As the Asian consensus on colorectal cancer screening suggested that screening for colorectal cancer should start at the age of 50 in Asia [92], IBS patients in Asia including India may undergo a colonoscopic examination if they are 50 years or older, particularly in the presence of alarm symptoms. However, more studies are needed from India on this issue before a firm recommendation can be made.

- **Statement 17:** The multi-dimensional clinical profile of IBS needs to be incorporated into clinical practice as proposed in the Rome IV algorithm

**Voting summary:** Accepted completely: (77.78%), accepted with minor reservation: (22.22%).

**Level of evidence:** II-2

**Grade of recommendation:** B

IBS is not a single disease but a syndrome with a constellation of multiple GI and non-GI symptoms, which might result from several pathophysiological mechanisms in variable combinations either in the gut or in the brain [17]. There are growing shreds of evidence to suggest a greater role of gut abnormalities than central pathophysiological mechanisms in the genesis of IBS symptoms [17]. Accordingly, the experts in the Rome IV Committee recommended underplaying the term “functional” to denote these disorders and re-name these conditions as the “Disorders of gut-brain interaction (DGBI)” [93]. It is important to note that in this new terminology that replaces FGID to denote these conditions, “gut” has been kept over “brain” to recognize the greater importance of gut-level pathophysiological mechanisms in the genesis of symptoms [17]. The Asian experts called these pathophysiological mechanisms micro-organic factors. Micro-organic factors such as slow colon transit and fecal evacuation disorder may be hidden behind the diagnosis of a patient with IBS-C. Similarly, IBS-D patients may have dietary intolerance, including that of lactose and fructose, bile acid malabsorption, non-celiac wheat sensitivity, SIBO and GI infection [11]. A recent study showed that multi-modality care targeting various pathophysiological mechanisms of IBS is superior to gastroenterologist-provided standard care [94]. Hence, unrevealing these pathophysiological mechanisms in each patient through thorough history taking, physical examination and investigation is a step towards personalized care of the patients with IBS. Multi-dimensional clinical profile (MDCP) is a step toward understanding the multiple factors contributing to symptom generation in patients with IBS (Table 6) [95]. This should be followed in clinical practice. MDCP assessment necessitates evaluation for the severity of IBS. Of different methods of severity assessment, IBS-SSS has been commonly used (Fig. 1) [79]. However, it has not yet been validated in Indian patients with IBS.

**Table 6:** Multi-dimensional clinical profile

Categorical diagnosis (symptom-based criteria) Clinical modifier (constipation- or diarrhea-predominant IBS, post-infection IBS, FODMAP sensitive) Impact (mild, moderate, severe) Psychosocial modifier Physiological dysfunction and biomarker	

*IBS* irritable bowel syndrome, *FODMAP* fermentable oligo-di-, monosaccharide and polyol

- **Statement 18:** Patients with refractory IBS symptoms need further pathophysiological evaluation

**Voting summary:** Accepted completely: (77.78%), accepted with minor reservation: (22.22%).

**Level of evidence:** II-1

**Grade of recommendation:** A

IBS is a syndrome in which the symptoms are contributed by multiple pathophysiological mechanisms [17]. Some of these pathophysiological factors differ in different subtypes of IBS. For example, in patients with IBS-C, the underlying physiological abnormalities may include, slow colon transit, fecal evacuation disorder or a variable combination of these two factors. Slow colon transit may result from excess methane production in the gut due to methanogen overgrowth. In contrast, the patients with non-C-IBS may have lactose and fructose malabsorption, bile acid malabsorption, non-celiac wheat and FODMAP intolerance, gut microbiota dysbiosis including SIBO, immune activation, and post-infection including post-COVID-19 etiology. These are, however, not watertight compartments; IBS-C patients may also have some of the pathophysiological factors listed under IBS-D and vice versa. Table 4 summarizes the major Indian studies substantiating these factors contributing to IBS [21, 22, 26–53]. Whereas the Asian workers suggested that IBS is a micro-organic condition to highlight the importance of these factors in the pathogenesis and management of the condition [1], the Rome IV experts brought in MDCP to give due importance to these factors (Table 6) [95].

- **Statement 19:** Counseling, reassurance and lifestyle modification are important in the management of IBS

**Voting summary:** Accepted completely: (88.89%), accepted with minor reservation: (11.11%).

**Level of evidence:** II-2

**Grade of recommendation:** B

The urban lifestyle associated with fad diets (junk, fast food, fatty food, tea, coffee, aerated drinks, etc.), substance abuse (smoking, chewing tobacco), low levels of physical activity, and psychological stress (depression, anxiety, insomnia) has been associated with higher prevalence of IBS [7, 13, 74, 96]. To inculcate lifestyle balance, remedial measures such as frequent counseling sessions and promoting physical activity (e.g. yoga, meditation) may help. Several randomized and non-randomized studies showed physical exercise, including yoga, is useful in the management of IBS [97–102]. A few studies compared yoga with either anti-anxiety medications or a low-FODMAP diet and found that yoga was either as good as anti-anxiety medications or was better than a low-FODMAP diet [103, 104]. Sleep disorders, common in IBS patients, should be recognized and appropriately treated with pharmacotherapy [105]. Melatonin has been found useful to treat sleep dysfunction [106]. Drugs with addictive potential such as benzodiazepines should be discouraged in a chronic condition like IBS.

Several randomized controlled trials showed the benefit of a low-FODMAP diet in the management of IBS [107]. However, the studies on low-FODMAP diet in India are scanty and are limited to one study on IBS and another on functional dyspepsia [42, 108, 109]. Though a recent review attempted to educate physicians about low-FODMAP diet in IBS [109], it may be somewhat premature in the bsence of more studies on efficacy and challenges of such treatment in India considering the high frequency of vegetarianism in the population [68]. An Australian study showed that multi-modality care involving a psychologist and dietician in addition to a gastroenterologist is superior to the standard gastroenterologist-provided treatment of IBS [94]. To ensure adequate compliance with lifestyle measures and dietary practices, patient education and counseling are the keys to success.

- **Statement 20:** The initial treatment of IBS is primarily symptom based

**Voting summary:** Accepted completely: (94.44%), accepted with minor reservation: (5.56%).

**Level of Evidence:** I

**Grade of Recommendation:** A

Since IBS is a syndrome diagnosed by symptom-based criteria, the goal of treatment of IBS is also to relieve patients’ symptoms and improve the quality of life (QOL). Currently, even the outcome of treatment of IBS also revolves around patient-reported outcome measures. Establishing a good doctor-patient relationship is an important art in the management of these patients. All bothersome symptoms should be targeted while treating these patients rather than only the predominant symptoms as suggested earlier taking into account specific IBS subtypes, symptom severity and contributing factors including psychosocial and dietary issues [63]. IBS patients with low symptom load do find significant improvement in their symptoms by use of antispasmodics [110, 111], bulking agents, antidiarrheals [112, 113], pro-motility agents including laxatives, [114, 115] and low-FODMAP diet [42]. An outline of the management of IBS is summarized in Fig. 2. Tables 7 and 8 list the drugs and their dosages used in different subtypes of IBS and some of the Indian studies.

- **Statement 21:** Antispasmodics are the first-line treatment of abdominal pain in patients with IBS and non-responsive patients may benefit from visceral neuromodulators

**Voting summary:** Accepted completely: (94.44%), accepted with minor reservation: (5.56%).

**Level of evidence:** I

**Grade of recommendation:** A

Several randomized controlled trials from Asia and meta-analyses showed antispasmodic drugs to be effective in the treatment of IBS, particularly when abdominal pain is a major symptom [1, 116–121]. These drugs also benefit exaggerated gastrocolic reflexes. The number needed to treat and harm with these drugs are quite good (5 and 17.5, respectively) [116]. However, there is hardly any good-quality study from India on antispasmodic drugs in the management of IBS. In a randomized controlled trial on 200 patients with Rome III criteria-diagnosed IBS, the authors found that drotaverine was superior to mebeverine in alleviating pain and stools-related symptoms [110]. It is important to mention that though some of the antispasmodic drugs such as anticholinergics are cheap, these are associated with significant adverse effects such as dry mouth and airway, visual blurring, palpitation, urinary dysfunction, constipation, and precipitation of angle-closure glaucoma. Hence, these drugs must be used with caution. Table 7 summarizes some of the studies from India on different antispasmodic drugs in the management of IBS [110, 111, 122, 123].

**Table 7:** Irritable bowel syndrome disease burden determines treatment with antispasmodics versus visceral neuromodulators

Authors	Treatment given	Response	Interpretation
Mishra et al. [122], 2014	Antispasmodic + probiotic Antispasmodic + probiotic + Amitriptyline Antispasmodic + probiotic + Riluzole	Category 3 showed best compliance	Central visceral neuromodulator (Riluzole) had superior efficacy than antispasmodic (Mebeverine) because disease burden was high
Rai et al. [110], 2014	Drotaverine hydrochloride (antispasmodic)	Significant improvement in abdominal symptoms as compared to placebo	Antispasmodic respond when disease burden is not high
Jayanthi et al. [123], 1998	Pinaverium bromide (open trial)	Significant relief in abdominal discomfort/pain and in bowel symptoms in most of the patients	Antispasmodic respond when disease burden is not high
Tiwari et al. [111], 2013	Bilvadileha (ayuvedic herbal drug)	Statistically significant improvement in all the clinical features of IBS as well as in the IBS severity score	Bilvadileha has antispasmodic property

Various psychoactive pharmacotherapy (currently called visceral neuromodulators) are useful to relieve abdominal pain in IBS patients working both through central and peripheral mechanisms [17, 124, 125]. These drugs may even work in the absence of significant psychological comorbidity [17].

- **Statement 22:** Laxatives and antidiarrheals are the first-line treatment for IBS-C and IBS-D, respectively

**Voting summary:** Accepted completely: (94.44%), accepted with minor reservation: (5.56%).

**Level of evidence:** I

**Grade of recommendation:** A

Bulking agents, including fibers, are quite popular to treat patients with IBS-C. However, there are inadequate high-quality studies to evaluate their efficacy. The fibers could be either water-soluble (e.g., psyllium, ispaghula, calcium polycarbophil, methylcellulose) or insoluble (e.g., corn, wheat bran). In an Indian dose-finding study, the authors found that the optimum dose of ispaghula husk, which is the husk of *Plantago ovata* seeds, was 20 g per day and it improved constipation and abdominal pain in IBS patients [113]. In a study from India on 20 patients, the authors concluded that the easing of bowel dissatisfaction appeared to be a major reason for the therapeutic success of ispaghula in IBS [112]. However, the fibers, particularly the insoluble ones, may aggravate abdominal bloating and flatulence [126, 127]. The other laxatives that may cause bloating and flatulence include lactulose and to some extent lactitol. Milk of magnesia and polyethylene glycol may cause minimum or no bloating. The stimulant drugs such as senna and bisacodyl are quite effective purgatives and do not cause abdominal bloating; these may cause abdominal cramps [128].

Loperamide and diphenoxylate are quite popular in the treatment of IBS-D. Several studies proved the efficacy of loperamide in the management of diarrhea but not abdominal pain or distension [129–133]. Other medicines that may be useful in patients with IBS-D include ramosetron and visceral neuromodulators with anticholinergic activity such as amitriptyline. Table 8 lists the drugs useful in treatment of different subtypes of IBS.

**Table 8:** Drugs used in different subtypes of irritable bowel syndrome

Drug class	Drug name	Therapeutic target	Dose	Remarks/adverse effects
**IBS-D**				
Opioid agonists (anti-motility agents)	Loperamide	μ-opioid receptor agonist	2–16 mg/d	
	Diphenoxylate		5–20 mg/d	Administered along with small doses of atropine to avoid addiction
	Eluxadoline	κ-, μ-opioid receptor agonist and δ-opioid receptor antagonist	75–100 mg twice daily	Pancreatitis due to sphincter of Oddi spasm Avoid in alcohol dependence and pre-existing pancreatico-biliary disease
Anti-serotonergics	Ramosetron	5HT3 receptor antagonist Retard colonic motility and reduce visceral pain	5 μg/d	
	Ondasetron		4 mg tid	Not useful for pain
	Alosetron		0.5–1 mg b.i.d	May cause severe constipation and ischemic colitis
Non-absorbable antibiotics	Rifaximin	Gut microbiota dysbiosis	550 mg thrice/day for 14 days	Retreatment effective in case of symptoms relapse
Bile acid sequestrants	Cholestyramine	Bind intraluminal bile acids	8–12 g/d (granules mixed in water)	Constipation
	Colestipol		2–16 g/d (tablet) 5–30 g/d (granules mixed in water)	
	Colesevelam		1875 mg/d (tablet)	
Probiotics		Gut microbiota dysbiosis		May help bloating Effect strain specific and dependent on dose
**IBS-C**				
Laxatives	Polyethylene glycol	Osmotic, bulking agent	17—34 g/day	Safe in bloater, unsafe in patients with renal failure
	Psyllium	Osmotic, bulking agents	20–30 g/day	Avoid in bloaters
	Magnesium hydroxide	Osmotic, bulking agents	30–60 mL/day	Avoid in bloaters
	Lactulose Lactitol	Osmotic and stool softener	15–30 mL/day for both	Avoid in bloaters
	Bisacodyl Sodium picosulfate	Stimulant Stimulates high-amplitude propagated contraction	5–15 mg/day	
	Senna	Stimulant	17.6–26.4 mg/day	
	Castor oil	Stimulant	15–60 mL/day	
	Liquid paraffin Docusate sodium	Stool softener	10–30 mL/day	
Secretagogue	Lubiprostone	Chloride channel activation Increase colonic secretion and transit	8 μg twice daily	Nausea dose dependent and may reduce by consuming with meals
	Linaclotide	Guanylate-C agonist Increase colonic secretion and transit	290 μg/d	
	Plecanatide		3 mg/d	
	Tenapanor	Sodium-hydrogen exchange 3 (NHE3) inhibitor Increase colonic secretion and transit	50 mg twice daily	
Enterokinetics	Prucalopride	5 HT4 agonist Increase colonic transit	1–2 mg/day	
	Tegaserod, Naronapride, Velusetrag			Tegaserod contraindicated in > 65y and with > 1 cardiovascular risk factors
	Cisapride (withdrawn) Mosapride (withdrawn)			Withdrawn due to cardiovascular adverse events
Bile acid absorption inhibitor	Elobixibat	Ileal bile acid transporter inhibitor (IBAT) Increase colonic bile acid levels, induce colonic secretion and motility	10 mg/d	
Non-absorbable antibiotics	Rifaximin	Action on methanogens	550 mg thrice/day for 14 days	Only useful in methane producers
Probiotics		Gut microbiota dysbiosis		May help bloating Effect strain specific and dependent on dose
**Miscellaneous drugs for IBS-C**				
	Glycerine Bisacodyl Suppository	Stool disimpaction	10 mg/day	
	Phosphate and citrate enemas	Stool disimpaction		
	Intra-sphincteric botulinum toxin injection	Fecal evacuation disorders	100 μg botulinum toxin into multiple sites into external sphincter and puborectalis	Shown to be useful alone, in combination with biofeedback and after biofeedback failure in patients with fecal evacuation disorder
**Abdominal pain**				
Antispasmodics	Clidinium bromide	Muscarinic Ach receptor inhibition	2.5 mg/d	Dry mouth, dizziness and blurred vision with centrally acting anticholinergics
	Mebeverine	Voltage gated L-type Ca channel inhibition	135 mg tid	
	Pinaverium		50 mg tid	
	Otilonium bromide	Voltage gated T- and L-type Ca channel inhibition, muscarinic Ach receptor inhibition, tachykinin NK2 antagonism	40 mg tid	
	Peppermint oil	Voltage gated L-type Ca channel inhibition, activate TRPM8 receptors on nociceptive afferents	187 mg tid	May worsen dyspepsia and heartburn
**Neuromodulators**				
Tricyclic anti-depressants (TCA)	Amitriptyline	Modulate norepinephrine and dopaminergic receptors Improves visceral and central pain	10–25 mg/d	Slow GI transit by anticholinergic effects and improve diarrhea in IBS-D Improves co-existing psychological distress Caution about anticholinergic side effects in elderly (dry mouth, eyes, urinary retention and cardiac arrhythmia)
	Nortriptyline		10–25 mg/d	
	Imipramine		25–75 mg/d	
	Desipramine		10–50 mg/d	
	Dosulepin		25–75 mg/d	
Selective serotonin reuptake inhibitors (SSRI)	Citalopram	Selectively increase 5-HT transmission by blocking presynaptic 5HT reuptake	10–20 mg/d	Increase GI transit and improve constipation in IBS-C Improves co-existing anxiety and depression Not helpful if pain is the dominant symptom
	Escitalopram		10–20 mg/d	
	Fluoxetine		10–20 mg/d	
	Sertraline		25–75 mg/d	
	Paroxetine		10–50 mg/d	
Serotonin noradrenaline reuptake inhibitors (SNRI)	Duloxetine	Increase 5-HT and NA transmission by blocking presynaptic 5HT and NA reuptake	30–60 mg/d	Side effect profile better than TCA and preferred when pain in the dominant symptom
	Venlafaxine		75–375 mg/d	
	Milnacipran		12.5–100 mg/d	

*IBS-D* irritable bowel syndrome-diarrhea predominant, *5-HT* serotonin, *IBS-C* irritable bowel syndrome-constipation predominant, *Ach* acetyl choline, *TRPM8* transient receptor potential melastatin 8, *NA* noradrenaline

- **Statement 23:** Dietary FODMAP restriction is useful in a proportion of IBS patients

**Voting summary:** Accepted completely: (94.44%), accepted with minor reservation: (5.56%), accepted with major reservation: (5.56%).

**Level of evidence:** I

**Grade of recommendation:** A

Dietary intolerance contributes to symptoms in patients with IBS [109]. A low-FODMAP diet in the management of IBS was first popularized in Australia. Subsequently, several randomized controlled trials and their meta-analysis proved the efficacy of a low-FODMAP diet in the management of IBS [134]. A low-FODMAP diet is particularly useful for flatulence, bloating, abdominal pain, distension and diarrhea [134]. However, studies on low-FODMAP diet in Indian patients with IBS are scanty. A randomized controlled trial on 166 patients showed that a strict low-FODMAP diet for the short-term and a modified low-FODMAP diet for the long-term may lead to symptom improvement in IBS-D patients [42]. Another randomized controlled study from the same group showed the efficacy of a low-FODMAP diet in another common form of FGID called functional dyspepsia [108]. However, considering the regional difference in dietary practices in India, there is a need for more studies to evaluate the efficacy, difficulty and patient acceptability of a low-FODMAP diet in different parts of India. The dietary variations, which may contribute to the challenges in the implementation of low-FODMAP diet in different regions of the country, may include the frequency of vegetarianism and lactose malabsorption, intake of milk in the diet, and the types of cereals commonly consumed (wheat vs. rice) [68]. In a recently published analysis of available data, it has been shown that implementing a low-FODMAP diet in northeastern India may be most easy compared to northern India, where it may be most difficult. Implementing a low-FODMAP diet in southern and eastern India may be easy, moderately easy in western India and not easy in central India [68].

- **Statement 24:** Probiotics may be helpful but more studies are needed

**Voting summary:** Accepted completely: (72.22%), accepted with minor reservation: (16.67%), accepted with major reservation: (11.11%).

**Level of evidence:** II-2

**Grade of recommendation:** B

Since gut microbiota dysbiosis and SIBO are associated with IBS, drugs manipulating gut microbiota such as antibiotics (rifaximin) and probiotics have been evaluated in its treatment. Another form of manipulation of gut microbiota using fecal transplantation is not recommended to treat IBS at present, but this therapy is only undertaken in a research setting. In an earlier consensus, the review of pieces of evidence suggested that *Lactobacillus* strains ameliorated flatulence significantly and abdominal or global symptom scores were improved by *Bifidobacterium* and *Escherichia* and *Streptococcus* strains both provided persistent symptom reduction [135]. However, the current evidences are not enough to achieve a strong consensus on the use of specific probiotics as a routine practice and its dose as the therapy of choice. More studies are needed on this issue from India.

- **Statement 25:** Non-constipated and methane-producing IBS-C patients benefit from rifaximin

**Voting summary:** Accepted completely: (55.56%), accepted with minor reservation: (33.33%), accepted with major reservation: (5.56%), reject with major reservation: (5.56%).

**Level of evidence:** I

**Grade of recommendation:** A

Several studies and meta-analyses showed that high-breath methane on lactulose hydrogen breath test was associated with constipation, due to slow colon transit [20, 21]. One retrospective and one prospective study from the USA showed that reduction in breath methane with antibiotic treatment improved constipation and a combination of neomycin and rifaximin was superior to rifaximin alone [23, 24]. A randomized controlled trial showed rifaximin was superior to a placebo in patients with slow transit constipation with excess breath methane on lactulose hydrogen breath test in association with the reduction in the breath methane and acceleration of colon transit [21]. In the previous Indian consensus on chronic constipation, the efficacy of rifaximin treatment for high-breath methane-producing constipation has been accepted [136]. Rifaximin has been proven to be effective in the treatment of non-C-IBS in a large clinical trial, which led to its approval by the Food and Drug Administration in the USA [137].

- **Statement 26:** Pathophysiology-directed therapy is important in the management of refractory IBS

**Voting summary:** Accepted completely: (83.33%), accepted with minor reservation: (11.11%), accepted with major reservation: (5.56%)

**Level of evidence:** **I**

**Grade of recommendation:** B

**Fig. 3:**
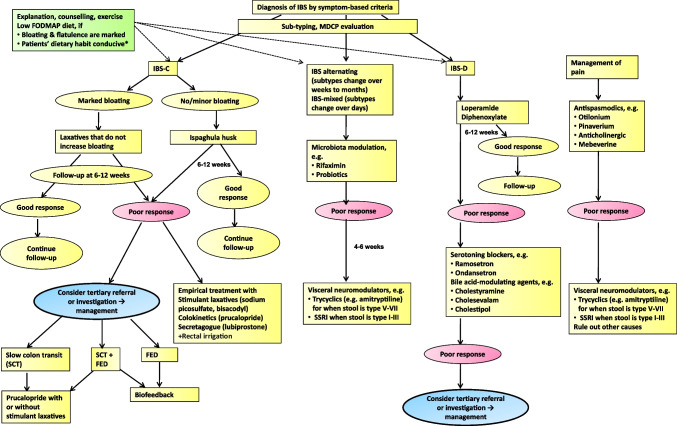
Algorithm of treatment of patients with irritable bowel syndrome. *Refer to reference [68] *IBS* irritable bowel syndrome, *MDCP* multi-dimensional clinical profile, *FODMAP* fermentable oligo-, di-, monosaccharide and polyol, *Refer to reference [68], *IBS-C* *D* *M* *U* constipation-, diarrhea-predominant, mixed, unclassified, *SCT* slow colon transit. *FED* fecal evacuation disorder, *SSRI* selective serotonin reuptake inhibitor

There are controversies in the definition of refractory IBS [138]. Refractory IBS patients either fail to respond appropriately to standard pharmacological treatment or show no improvement with conventional pharmacological interventions and continue to have severe symptoms. In the current consensus, if the symptoms persist or increase in severity even during 12 weeks of follow-up, the condition may be considered clinically refractory, albeit empirically. These patients should be referred to a center with facilities for further specialized investigations and multi-modality care not only by the gastroenterologist alone but by a GI disorder experienced psychologist/psychiatrist and dietician. These patients should be assessed clinically for symptom duration, symptom severity (IBS-SSS) (Figs. 1 and 2), symptom type, dietary practices and psychological issues. The stubbornness of refractory IBS may be explained by multi-dimensional pathophysiological mechanisms, including altered gut motility, intestinal barrier dysfunction, gut microbiota dysbiosis, including SIBO, gut immune dysfunction, visceral hypersensitivity, bile acid malabsorption, FODMAP sensitivity, psychological factors and altered gut-brain interactions [11, 17, 139, 140]. Investigations directed to these pathophysiological mechanisms, based on the local availability, should be undertaken (Figs. 2 and 3). Both pharmacological and non-pharmacological treatment directed to each of the pathophysiological mechanisms, even if needed combination treatment is recommended to treat patients with refractory IBS as reviewed previously [11, 17, 141]. Pharmacological (Table 8) and non-pharmacological interventions (e.g. cognitive behavioral therapy, gut-directed hypnotherapy, mindfulness-based stress reduction therapy [MBSRT]) directed against abnormal gut-brain interaction using visceral neuromodulators and various forms of psychotherapies have been found useful in the treatment of refractory IBS [125] (Fig. 3). A multi-modality care involving a psychologist and dietician in addition to a gastroenterologist is expected to be superior to the standard gastroenterologist-provided treatment of refractory IBS [94].

- **Statement 27:** Psychological interventions are useful in those with psychiatric comorbidities or refractory IBS

**Voting summary:** Accepted completely: (83.33%), accepted with minor reservation: (11.11%), rejected with minor reservation: (5.56%)

**Level of evidence:** II-1

**Grade of recommendation:** B

Psychological comorbidity is common among IBS patients, particularly those presenting to tertiary care facilities [62]. Patients with severe symptoms, non-responding patients and overlap disorders more often have psychological comorbidity [17]. Psychological interventions either with pharmacotherapy or cognitive behavior therapy, gut-directed hypnotherapy, yoga and MBSRT are useful options to treat these patients in addition to standard treatment [142, 143]. It is important to mention here that while using different visceral neuromodulator/ psychoactive compounds, one should use those with prominent anticholinergic side effects in IBS-D patients and serotonin reuptake inhibitors (SSRIs) in IBS-C patients for additional benefit towards amelioration of GI symptoms (Table 8).

Published experience in non-pharmacological psychological intervention on IBS in India is limited. These interventions are also not available widely. In a non-randomized open-label controlled study on MBSRT on 47 IBS patients (30 on MBSRT and 17 pharmacological treatment alone), MBSRT led to a greater improvement in the quality of life and mindfulness components and reduced IBS symptoms as compared with the control group [144]. One Indian study also showed the efficacy of yoga in the treatment of IBS [101].

- **Statement 28:** Overlapping functional GI disorders may require combination treatment

**Voting summary:** Accepted completely: (94.44%), accepted with minor reservation: (5.56%),

**Level of evidence:** **II-2**

**Grade of recommendation:** B

IBS may occur concomitantly with other FGIDs, especially, IBS-functional dyspepsia overlap [7, 13, 17] and IBS-GERD overlap [145]. Table 3 presents the IBS-FGID overlap in studies conducted entirely or partly in India. A recent Asia Pacific Consensus reviewed the clinical spectrum, pathophysiology and the management of overlapping FGIDs in details. The authors in this consensus did suggest either pathophysiology-guided or symptom-directed combination treatment to manage these patients. However, it is important to remember for possible drug interaction while combining different drugs in the management of IBS.
